# Understanding how discrete populations of hypothalamic neurons orchestrate complicated behavioral states

**DOI:** 10.3389/fnsys.2015.00111

**Published:** 2015-08-04

**Authors:** Allison K. Graebner, Manasi Iyer, Matthew E. Carter

**Affiliations:** Program in Neuroscience, Department of Biology, Williams CollegeWilliamstown, MA, USA

**Keywords:** hypothalamus, Hcrt, hypocretin, orexin, AgRP, OVLT, optogenetics, pharmacogenetics

## Abstract

A major question in systems neuroscience is how a single population of neurons can interact with the rest of the brain to orchestrate complex behavioral states. The hypothalamus contains many such discrete neuronal populations that individually regulate arousal, feeding, and drinking. For example, hypothalamic neurons that express hypocretin (Hcrt) neuropeptides can sense homeostatic and metabolic factors affecting wakefulness and orchestrate organismal arousal. Neurons that express agouti-related protein (AgRP) can sense the metabolic needs of the body and orchestrate a state of hunger. The organum vasculosum of the lamina terminalis (OVLT) can detect the hypertonicity of blood and orchestrate a state of thirst. Each hypothalamic population is sufficient to generate complicated behavioral states through the combined efforts of distinct efferent projections. The principal challenge to understanding these brain systems is therefore to determine the individual roles of each downstream projection for each behavioral state. In recent years, the development and application of temporally precise, genetically encoded tools has greatly improved our understanding of the structure and function of these neural systems. This review will survey recent advances in our understanding of how these individual hypothalamic populations can orchestrate complicated behavioral states due to the combined efforts of individual downstream projections.

To ensure that an animal obtains an optimal amount of sleep, food, and water, the brain must sense the internal environment and influence behavior by producing sensations we describe as “tired/awake, “hungry/full, and “thirsty/quenched.” Amazingly, these complicated behavioral states are often controlled by discrete populations of neurons, some composed of only 1000–2000 individual cells. The hypothalamus contains many such populations including hypocretin (Hcrt—also called “orexin”)-expressing neurons in the lateral hypothalamus that regulate wakefulness and arousal (Alexandre et al., 2013), agouti-related peptide (AgRP)-expressing neurons in the arcuate nucleus that regulate hunger (Liu et al., 2013), and organum vasculosum of the lamina terminalis (OVLT) neurons in the preoptic area that regulate thirst (Bourque, 2008). Each is able to sense signals from the internal environment and influence animal behavior through diffuse projections throughout the brain.

A term often used to describe the actions of these neuronal populations is “orchestrate”—the implication being that these neurons mobilize and unify the actions of several discrete players to achieve an outcome that is more than the sum of its parts. Just as a musical conductor directs an orchestra to produce a recognizable piece of music, neural activity in these discrete populations of neurons orchestrates recognizable behavioral phenotypes. For example, stimulation of Hcrt neurons produces an increase in wakefulness, while stimulation of AgRP neurons produces an immediate increase in food-seeking behavior. The exciting scientific challenge ahead is to determine exactly how these neurons orchestrate such deliberate, complicated behavioral states by dissecting the functional contributions of their downstream projections.

When investigating the functional roles of various downstream projections from a population of neurons, what phenotypes should we hypothesize to observe? Surely, some individual projections may reproduce fundamental aspects of the overall behavior, while others may produce phenotypes that the investigator may not even initially consider examining. For example, neural populations that orchestrate global wake states may also engage circuitry involved in stress. Populations that orchestrate hunger may also engage circuitry that suppresses growth and fertility. Populations that orchestrate thirst may also engage circuitry that regulates sodium appetite. A thorough investigation of how these neural populations influence complex behavioral states must consider several distinct elements that each contributes to a grander, global phenotype.

These considerations are particularly important given the recent development and application of genetically encoded neuronal actuators that have allowed for the unprecedented study of discrete neuronal populations and their downstream projections. Optogenetic technology has allowed scientists the ability to stimulate or inhibit genetically-defined populations of neurons with millisecond-precise temporal resolution (Yizhar et al., 2011). Pharmacogenetic technology, such as the “Designer Receptors Exclusively Activated by Designer Drugs”—DREADDS, allows for the ability to hijack a neuron’s G-protein signaling cascade to induce or suppress neural activity (Armbruster et al., 2007). Importantly, these technologies not only allow for the manipulation of neural activity at the soma, but also in distal axonal projections (Fenno et al., 2011; Stachniak et al., 2014). Thus, these technologies have allowed for the remarkable opportunity to investigate the contribution of each downstream projection in an overall behavioral phenotype.

Below, we consider the functional dissection of downstream projections for Hcrt neurons in arousal, AgRP neurons in hunger, and OVLT neurons in thirst. For each population of neurons, we provide a brief history of the study of these neurons and highlight the potential downstream circuit elements that amalgamate to cause a complex behavioral state.

## Hcrt Neurons Orchestrate Wakefulness and Arousal

### Defining Wakefulness and Arousal

Wakefulness is defined as a conscious state in which an animal can perceive and interact with its environment. This behavioral state is distinct from sleep, which is defined as a rapidly reversible state of immobility and greatly reduced sensory responsiveness to environmental stimuli (Siegel, 2008). In mammals, sleep is generally divided into slow wave sleep (SWS, or NREM sleep) and rapid eye movement (REM) sleep. Wakefulness, SWS, and REM sleep can be quantitatively defined by precise electroencephalographic (EEG) and electromyographic (EMG) features.

Although these states of sleep and wake are qualitatively and quantitatively easy to characterize, it is surprisingly difficult to define what is meant by “arousal.” This term usually describes the degree of vigilance during wakefulness, manifested by an increased length of wake bouts, locomotor activity, stress hormone activity, cardiovascular activity, thermogenesis, respiration, and tendency to engage in behaviors such as sexual interactions, eating, and drug seeking. Simultaneously, heightened arousal usually results in decreased symptoms of depression, sensitivity to pain, and the release of growth hormone.

### The Hypocretin System

Although multiple populations of neurons in the brain are thought to regulate wakefulness and arousal, much research has focused on a population of neurons in the lateral hypothalamus that express Hcrt neuropeptides (Hcrt neurons; Saper et al., 2001, 2005; Sutcliffe and de Lecea, 2002; Sakurai, 2007; Carter et al., 2009b). The Hcrts consist of a pair of two neuropeptides, Hypocretin-1 and Hypocretin-2 (Hcrt1 and Hcrt2; also known as Orexin A and Orexin B, respectively) that are processed from the same genetic precursor, “preprohypocretin” (de Lecea et al., 1998; Sakurai et al., 1998). In the brain, these peptides are expressed exclusively in the perifornical lateral hypothalamus (de Lecea et al., 1998; Sakurai et al., 1998). Hcrt neurons are glutamatergic and cause excitatory effects on postsynaptic targets (de Lecea et al., 1998; van den Pol et al., 1998; Date et al., 1999; Rosin et al., 2003). Interestingly, these neurons express other neuromodulators, such as dynorphin (Chou et al., 2001). Hcrt neurons are completely distinct from an intermingled population of neurons in the lateral hypothalamus that express melanin-concentrating hormone (MCH; Broberger et al., 1998a).

Hcrt neurons receive afferent projections from many nuclei throughout the brain including from neurons that are glutamatergic and GABAergic (Henny and Jones, 2006). Consistent with their known role in promoting wakefulness, Hcrt neurons are excited by neurotransmitters that promote arousal including ATP (Wollmann et al., 2005), corticotropin releasing factor (Winsky-Sommerer et al., 2004), thyrotropin releasing hormone (Hara et al., 2009), noradrenaline (Bayer et al., 2005), and acetylcholine (Bayer et al., 2005; Henny and Jones, 2006). Importantly, sleep-promoting molecules inhibit Hcrt neurons including GABA and adenosine (Thakkar et al., 2002; Liu and Gao, 2007).

In turn, Hcrt neurons project diffusely throughout the brain. Intracerebroventricular (i.c.v.) injection of Hcrt neuropeptides into the brain causes an increase in expression of Fos, an indirect marker of neural activity, widely throughout the brain (Date et al., 1999). Hcrt neurons send especially dense projections to the locus coeruleus (LC), tuberomammilary nucleus (TMN), ventral tegmental area (VTA), dorsal raphe nuclei, other hypothalamic nuclei, and the cortex (Peyron et al., 1998; Date et al., 1999). The afferent projection patterns of Hcrt neurons match the expression patterns of the two hypocretin receptors, Hcrt-r1 and Hcrt-r2 (Trivedi et al., 1998). Hcrt-r1 binds Hcrt1 with high affinity and binds Hcrt with 100–1000-fold lower affinity; Hcrt-r2 has a high affinity for both Hcrt1 and Hcrt2 (Sakurai et al., 1998; Lang et al., 2006).

Neural activity in Hcrt neurons correlates with states of heightened arousal. Expression of Fos in Hcrt neurons correlates with wakefulness (Estabrooke et al., 2001) and release of Hcrt1 is significantly higher during wakefulness compared to sleep (Kiyashchenko et al., 2002). *In vivo* recordings in freely moving animals show that Hcrt neurons fire maximally during arousal, including behavior accompanied with strong locomotor activity such as eating, grooming, and exploratory behavior (Lee et al., 2005; Mileykovskiy et al., 2005). In contrast to their spontaneous activity in acute brain slices (Eggermann et al., 2003), Hcrt neurons are relatively silent during periods of low activity, completely silent during SWS and REM sleep, and are reactivated during REM sleep-to-wake transitions (Lee et al., 2005; Mileykovskiy et al., 2005). In fact, bursts of activity in Hcrt neurons during sleep predict sleep-to-wake transitions (Lee et al., 2005).

The crucial role of hypocretins in arousal stability stems from the original finding that impairment of the Hcrt system causes the sleep disorder narcolepsy in mice (Chemelli et al., 1999; Hara et al., 2001; Mochizuki et al., 2004), dogs (Lin et al., 1999), and humans (Nishino et al., 2000; Peyron et al., 2000; Gencik et al., 2001). Most human narcoleptic patients have decreased levels of Hcrt in their cerebrospinal fluid (Nishino et al., 2000; Ripley et al., 2001; Dauvilliers et al., 2003) and postmortem analysis shows a reduction of Hcrt neurons (Thannickal et al., 2000) and reduced expression of the Hcrt peptides (Peyron et al., 2000) in the brain. Interestingly, Hcrt levels are also reduced in other diseases associated with deficits in wakefulness including Guillain-Barre syndrome (Ripley et al., 2001; Kanbayashi et al., 2002) and myotonic dystrophy (Martínez-Rodríguez et al., 2003), while Hcrt levels are elevated in some patients with restless leg syndrome (Allen et al., 2002). The Hcrt system is also necessary for the increase in arousal typically observed in response to fasting (Yamanaka et al., 2003), as well as for normal emergence from general anesthesia (Kelz et al., 2008). Using modern optogenetic techniques, it has been shown that photoinhibition of Hcrt neurons expressing the inhibitory chloride pump Halorhodopsin from Natronomonas (NpHR) induces slow wave sleep (Tsunematsu et al., 2011). Taken together, these studies demonstrate the necessity of Hcrt neurons in maintaining and promoting normal states of wakefulness.

Gain of function studies demonstrate that Hcrt peptides and Hcrt neurons are sufficient to increase wakefulness. I.c.v. injection of Hcrt1 increases wakefulness (Piper et al., 2000) as well as general locomotor behaviors such as burrowing, grooming, and exploratory activity (Ida et al., 1999; Furlong et al., 2009). Optogenetic photostimulation of Hcrt neurons expressing Channelrhodopsin 2 (ChR2) causes sleep-to-wake transitions throughout the light/dark cycle (Adamantidis et al., 2007; Carter et al., 2009a). Likewise, pharmacogenetic stimulation of Hcrt neurons with the artificial (DREADD) stimulatory hM_3_Dq receptor increases wakefulness and reduces sleep.

### Hcrt Neuron Projections Mediating Wakefulness

How do Hcrt neurons orchestrate wakefulness and arousal? Hcrt neurons project to many nuclei throughout the brain known to promote arousal, such as the LC, TMN, VTA and basal forebrain (Saper et al., 2005). Indeed, Hcrt neuropeptides cause depolarization of postsynaptic cells and an increase in wakefulness when selectively microinjected into the LC (Hagan et al., 1999; Horvath et al., 1999; Ivanov and Aston-Jones, 2000; Walling et al., 2004), TMN (Bayer et al., 2001; Eriksson et al., 2001; Huang et al., 2001; Ishizuka et al., 2002; Schöne et al., 2012), basal forebrain (BF; Eggermann et al., 2001; España et al., 2001; Thakkar et al., 2001), dorsal raphe nucleus (Brown et al., 2001, 2002; Liu et al., 2002; Takahashi et al., 2005), VTA (Nakamura et al., 2000; Korotkova et al., 2003), laterodorsal tegmental nucleus (Xi et al., 2001; Burlet et al., 2002), preoptic area (Methippara et al., 2000), and pontine reticular formation (Brevig et al., 2010). Thus, several downstream brain regions are sufficient to mediate, at least in part, Hcrt neurons effects on wakefulness.

Whether any single downstream region is necessary for Hcrt neurons to promote wakefulness is a matter of debate. Huang et al. (2001) showed that i.c.v. injection of Hcrt increases wakefulness, but not in H1R knockout mice that don’t express receptors for histaminergic signaling. Because histamine in the brain is selectively produced by neurons in the TMN, this study raises the possibility that the TMN is necessary for Hcrt-mediated sleep to wake transitions. However, optogenetic photostimulation of Hcrt neurons causes sleep-to-wake transitions in histidine decarboxylase knockout mice that lack the ability to produce histamine (Carter et al., 2009a), calling into question the necessity of histaminergic signaling for Hcrt-mediated wakefulness. In contrast, in wild type mice, Hcrt-mediated sleep-to-wake transitions are blocked when the LC is concomitantly inhibited (Carter et al., 2012). Furthermore, knockdown of the Hcrt-r1 receptor in the LC increases REM sleep (Chen et al., 2010). Knockdown of the Hcrt-r2 receptor in the pontine reticular formation also increases REM sleep and can cause behavioral cataplexy (Thakkar et al., 1999). Additionally, Hcrt-induced hyperlocomotion and grooming behavior is significantly reduced following central administration of dopamine receptor antagonists (Nakamura et al., 2000), suggesting the necessity of the VTA-dopamine system in mediating Hcrt’s effects of hyperarousal during wakefulness. Thus, the effect of Hcrt neurons on wakefulness probably involves more than one downstream target, with different postsynaptic neurons playing an important, distinct role.

Informative attempts have been made to rescue muscle paralysis and normal bouts of wakefulness in mouse models of narcolepsy by selectively expressing Hcrt peptides or Hcrt receptors in specific brain regions. Hcrt gene transfer into the zona incerta (Liu et al., 2011) or dorsolateral pons (Blanco-Centurion et al., 2013) of Hcrt knockout mice is sufficient to reduce cataplexy and improve wake maintenance. In mice lacking Hcrt receptors, selective re-expression of receptors in the dorsal raphe nucleus or LC attenuates cataplexy and fragmentation of wakefulness (Hasegawa et al., 2014).

### Hcrt Neuron Projections Mediating Arousal-Related Behaviors

Beyond simple classifications of sleep and wake, environmental conditions and stimuli that increase arousal also often engage the physiological response to stress. Consistently, Hcrt neurons project to corticotropin releasing factor (CRF) neurons within the paraventricular nucleus (PVN; Peyron et al., 1998), neurons that activate the hypothalamus-pituitary-adrenal (HPA) axis which regulates circulating stress hormones. Centrally administered Hcrt increases activity in CRF-expressing PVN neurons (Kuru et al., 2000; Samson et al., 2002; Sakamoto et al., 2004) and causes a dose-dependent increase in plasma concentration of adrenocorticotropic hormone (ACTH) and corticosterone (Jászberényi et al., 2000; Kuru et al., 2000; Al-Barazanji et al., 2001; Russell et al., 2001; Samson et al., 2002). Behaviorally, Hcrt neurons may induce anxiety-like behavior through projections to the bed nucleus of the stria terminalis (BNST; Lungwitz et al., 2012) and central amygdala (Bisetti et al., 2006). Taken together, these reports demonstrate the necessity and sufficiency of Hcrt signaling in the physiological and behavioral hallmarks of stress, and point to the PVN, BNST, and amygdala as critical sites of action.

Hcrt neurons have also been shown to engage physiological aspects of a heightened arousal state. Central injection of Hcrt peptides causes an increase in heart rate and mean arterial pressure (Samson et al., 1999; Shirasaka et al., 1999; Antunes et al., 2001; Wang et al., 2001) as does selective microinjection into the rostroventral lateral medulla (RVLM; Chen et al., 2000; Machado et al., 2002; Shahid et al., 2012), nucleus of the solitary tract (NTS; de Oliveira et al., 2003), raphe nuclei (Luong and Carrive, 2012), and intrathecal cavity of the spinal cord (Shahid et al., 2011). Ablation of Hcrt receptors or antagonism of Hcrt receptors reduces basal blood pressure (Kayaba et al., 2003; Li et al., 2013). Central injection of Hcrts also causes an increase of oxygen consumption (Wang et al., 2001; Asakawa et al., 2002; Young et al., 2005), an effect mimicked by selective microinjection into the preBötzinger or phrenic nuclei (Young et al., 2005). Finally, central injection of Hcrts causes an increase in brown adipose tissue thermogenesis and body temperature (Balaskó et al., 1999; Yoshimichi et al., 2001; Monda et al., 2007), an effect mediated by the ventromedial hypothalamus (VMH; Monda et al., 2005), and raphe pallidus (Tupone et al., 2011). Taken together, these studies not only demonstrate a clear role for Hcrt neurons in promoting physiological hallmarks of arousal, but also specific anatomical targets that mediate their effects.

Hcrt neurons also play a role in food-seeking behavior. Indeed, the alternate name for Hcrts, orexins, was termed due to the original finding that i.c.v. infusion of Hcrts increase food intake (Sakurai et al., 1998). However, this effect is not as potent as other orexigenic peptides (Edwards et al., 1999; see below for a description of orexigenic AgRP neurons). Antagonism of Hcrt receptors enhances behavioral satiety (Rodgers et al., 2001), and food anticipatory activity is decreased when Hcrt neurons are genetically ablated (Akiyama et al., 2004). The effects of Hcrt neurons on food-seeking behavior seem to be mediated by direct projections to the arcuate nucleus (Burdakov et al., 2003; van den Top et al., 2004; Ma et al., 2007), a key brain region that regulates energy homeostasis, as well as the lateral hypothalamus and paraventricular hypothalamus (PVH; Dube et al., 1999; Sweet et al., 1999).

A heightened arousal state also often leads to alterations in reward processing, such as reinstatement of previously extinguished drug-seeking behavior or increased association between an environmental cue and drug reward. Interestingly, Hcrt neurons have been repeatedly shown to play a major role in cue-induced reinstatement and reward seeking in response to cocaine (Boutrel et al., 2005; Harris et al., 2005; Borgland et al., 2006; Harris and Aston-Jones, 2006; Smith et al., 2009, 2010), ethanol (Shoblock et al., 2011; Srinivasan et al., 2012), nicotine (Hollander et al., 2008; LeSage et al., 2010; Plaza-Zabala et al., 2010), morphine (Georgescu et al., 2003; Harris et al., 2005, 2007; Narita et al., 2006; Sharf et al., 2008, 2010), and heroin (Smith and Aston-Jones, 2012). These effects are largely mediated by direct projections to the mesolimbic VTA (Borgland et al., 2006, 2008; España et al., 2010, 2011; Srinivasan et al., 2012; Taslimi et al., 2012; Hrabovszky et al., 2013) and nucleus accumbens (Thorpe and Kotz, 2005; Sharf et al., 2008; Mukai et al., 2009; Mori et al., 2011), brain structures well known for their roles in regulating reward processing.

Recently, an interesting debate has emerged about the potentially dichotomous functions of Hcrts in wakefulness and reward seeking. Harris and Aston-Jones (2006) suggested that Hcrt neurons can be functionally subdivided into a medial group, which regulates wakefulness and stress, and a lateral group, which regulates reward. This hypothesis was initially supported by the finding that hypocretin neurons can be functionally subdivided into at least two populations based on their electrophysiological properties (Schöne et al., 2011). However, retrograde tracers injected into either the LC or VTA do not seem to preferentially label either electrically-active class of Hcrt neuron, nor do they preferentially label a medial or lateral population (González et al., 2012). Interestingly, no study has injected retrograde tracers into two downstream populations of Hcrt neurons in the same animal, thus, future studies are needed to resolve whether Hcrt neurons can be anatomically and functionally subdivided into two or more populations.

Hcrt neurons also promote hyperarousal during sexual behavior. Hcrt neurons potentiate male sexual behavior (Di Sebastiano et al., 2010), and antagonism of Hcrt receptors blocks copulation (Muschamp et al., 2007) and prevents conditioned place preference for sexual behavior (Di Sebastiano et al., 2011). These effects seem to be mediated by direct projections to the median preoptic area (MPOA; Gulia et al., 2003).

A state of behavioral arousal is not only associated with increases in activity and physiological parameters, but also an active suppression of parameters such as depression, pain, and growth. Interestingly, Hcrt neurons have been shown to induce an antidepressive-like effect in rodent models of depression (Feng et al., 2008), potentially mediated by projections to the VTA (Nocjar et al., 2012) and hippocampus (Ito et al., 2008). Hcrt neurons have been repeatedly demonstrated to exert analgesic and anti-nociceptive properties (Bingham et al., 2001), effects mediated by direct projections to pain-processing areas such as the ventrolateral periaqueductal gray (vlPAG; Azhdari Zarmehri et al., 2011; Ho et al., 2011) and laminae I-II neurons in the spinal cord (Yamamoto et al., 2002, 2003; Cheng et al., 2003; Suyama et al., 2004; Kajiyama et al., 2005; Mobarakeh et al., 2005). Finally, Hcrt neurons suppress the release of growth hormone releasing hormone (GHRH), and therefore indirectly the release of plasma growth hormone (Seoane et al., 2004), by projections to GHRH-expressing cells in the PVH (López et al., 2004).

**Figure 1:**
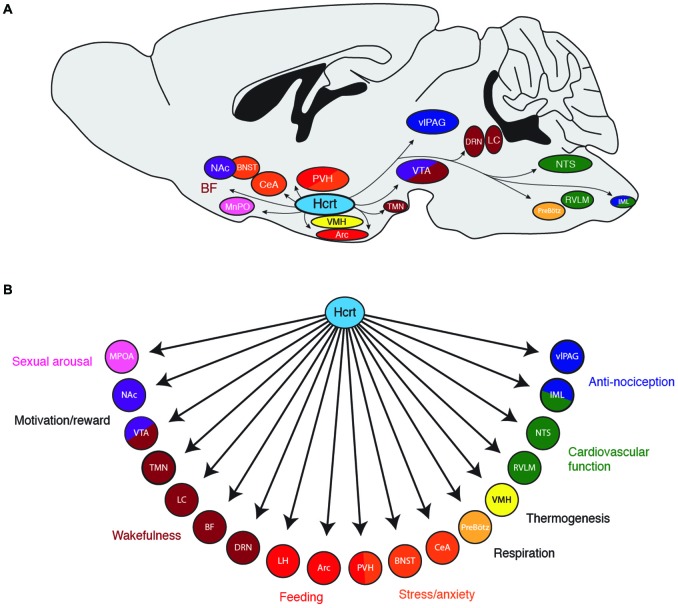
**(A)** Downstream projections from hypocretin (Hcrt) neurons and their **(B)** potential functional roles in wakefulness and arousal. Arc, arcuate nucleus; BF, basal forebrain; BNST; bed nucleus of the stria terminalis; CeA, central nucleus of the amygdala; DRN, dorsal raphe nuclei; IML, intermediolateral cell column of the spinal cord; LC, locus coeruleus; LH, lateral hypothalamus; MPOA, median preoptic area; NAc, nucleus accumbens; NTS, nucleus of the solitary tract; PreBötz, preBötzinger complex; PVH, paraventricular hypothalamus; RVLM, rostroventral lateral medulla; TMN, tuberomammilary nucleus; vlPAG, ventrolateral periaqueductal gray; VMH, ventromedial hypothalamic nucleus; VTA, ventral tegmental area.

Taken together, these studies demonstrate that Hcrt neurons can orchestrate a behavioral state of wakefulness and various forms of arousal via widespread projections throughout the brain (Figure 1). Like examining the contribution of a single section of an orchestra, investigating the contribution of Hcrts on any single brain region will not reflect the global sum of Hcrt activity on heightened states of arousal. An important question for future research is whether Hcrt neurons can be anatomically and functionally subdivided into multiple, discrete subpopulations, each projecting to a distinct downstream region. Additionally, it will be beneficial to know if different patterns of neural activity in Hcrt neurons (e.g., different firing frequencies or temporal patterns of action potentials) selectively recruit different downstream nuclei due to differential expression of postsynaptic receptors.

## AgRP Neurons Orchestrate Food-Seeking Behavior

### AgRP Neurons and Feeding

The brain regulates food intake by detecting peripheral signals about nutrient status and energy stores, integrating this information with cognitive and sensory cues, and producing appropriate feeding behavior (Schwartz et al., 2000; Abizaid et al., 2006; Morton et al., 2006). While there are multiple populations of neurons in the brain that help regulate feeding, much previous research has focused on orexigenic AgRP neurons in the hypothalamic arcuate nucleus as being principal mediators of food seeking behavior. In addition to expressing AgRP, these neurons also co-express neuropeptide Y (NPY; Broberger et al., 1998b; Hahn et al., 1998) as well as the inhibitory neurotransmitter GABA (Tong et al., 2008; Wu et al., 2009). AgRP neurons are distinct from an intermingled population of neurons in the arcuate nucleus that express pro-opiomelanocortin (POMC; Hahn et al., 1998; Wilson et al., 1999).

AgRP neurons directly sense and respond to circulating satiety and hunger signals. For example, AgRP neurons express receptors for leptin, a satiety hormone secreted by fat cells that causes a decrease in food-seeking behavior (Halaas et al., 1995; Pelleymounter et al., 1995). In response to leptin, these neurons downregulate AgRP and NPY mRNA expression and decrease neuronal activity (Mizuno and Mobbs, 1999; Korner et al., 2001; van den Top et al., 2004; Baver et al., 2014). In contrast, AgRP neurons also express receptors for ghrelin, a hunger-inducing hormone secreted by the stomach (Nakazato et al., 2001). In response to ghrelin and conditions of fasting, these neurons upregulate AgRP and NPY mRNA expression and increase neuronal activity (Kamegai et al., 2000; Nakazato et al., 2001; Seoane et al., 2003; van den Top et al., 2004; Takahashi and Cone, 2005; Staszkiewicz et al., 2007; Wang et al., 2014). Thus, AgRP neurons are primed to detect peripheral signals from the body and orchestrate a central feeding response.

Loss of function experiments show that AgRP neurons are necessary for normal feeding behavior. Genetic ablation of AgRP neurons results in mice that are hypophagic and lean (Bewick et al., 2005; Gropp et al., 2005), or even mice that stop eating altogether (Luquet et al., 2005). Interestingly, genetic ablation of only the AgRP or NPY peptides (or both in combination) do not result in a lack of feeding (Qian et al., 2002), suggesting a critical role for GABA in mediating the downstream effects of AgRP neurons. Indeed, selective removal of GABA from AgRP neurons causes decreased feeding (Tong et al., 2008; Krashes et al., 2013), an effect that can be rescued by delivering a GABA agonist into the brain (Wu et al., 2009).

Consistent with loss of function experiments, gain of function studies show that AgRP neurons are sufficient to induce feeding. Central injection of the AgRP peptide promotes feeding (Rossi et al., 1998) and chronic overexpression of AgRP causes obesity (Graham et al., 1997; Ollmann et al., 1997). The AgRP peptide directly antagonizes the MC3R and MC4R melanocortin receptors (Ollmann et al., 1997; Quillan et al., 1998; Rossi et al., 1998), critical components of the melanocortin system that promotes satiety and decreases feeding (Cone, 2005). Recently, genetically-encoded neuromodulation tools have been used to selectively stimulate AgRP neural activity in freely-behaving mice. ChR2 mediated-stimulation of AgRP neurons causes immediate feeding (Aponte et al., 2011), while hM3Dq-mediated stimulation of AgRP neurons causes robust feeding over hours and an obesity phenotype when applied over several days (Krashes et al., 2011). Taken together, these studies show that AgRP neurons can selectively orchestrate a specific behavioral state of robust feeding.

### AgRP Neuron Projections that Mediate Feeding

To determine how AgRP neurons orchestrate feeding, previous studies selectively investigated the effect injecting the AgRP peptide directly into discrete target regions on food intake. AgRP neurons project to the PVN, the BNST, the PAG, the parabrachial nucleus (PBN), the lateral septum (LS), the median preoptic nucleus (MnPO), the central nucleus of the amygdala (CeA), the dorsomedial hypothalamic nucleus (DMH), the ventromedial hypothalamic nucleus (VMH), and the POMC-expressing neurons in the arcuate nucleus (Broberger et al., 1998b; Haskell-Luevano et al., 1999; Légrádi and Lechan, 1999; Mihály et al., 2000). Selective injection of the AgRP peptide into the PVN, DMN, MPO, and CeA increases feeding (Cowley et al., 1999; Kim et al., 2000; Wirth and Giraudo, 2000; Boghossian et al., 2010), whereas injections into the arcuate, lateral hypothalamus, and VMH has no effect (Kim et al., 2000). However, because AgRP is not the only peptide/transmitter released by these neurons, these studies do not rule out the possibility that GABA mediates an increase in feeding in these areas.

Recently, two laboratories employed different methods to investigate which downstream projections of AgRP neurons mediate an increase in food intake. The lab of Richard Palmiter generated *AgRP^DTR^* mice in which the diphtheria toxin receptor (DTR) is selectively expressed in AgRP neurons, causing selective ablation of these neurons when mice are injected with diphtheria toxin. Ablation of AgRP neurons causes a starvation phenotype (Luquet et al., 2005) and, consistent with their expression of the inhibitory neurotransmitter GABA, an increase in Fos expression in downstream targets (Wu et al., 2008). To determine if increasing inhibition to one or more of these downstream targets could rescue feeding in these mice, the GABA_A_ receptor agonist bretazenil was selectively injected into the LS, PAG, PVH, and PBN. Surprisingly, bretazenil injection into the PBN (but not other areas) rescued feeding after AgRP ablation. Subsequent work showed that the neurons that mediated this effect are located in the external lateral region of the PBN and specifically express calcitonin gene related peptide (CGRP; Carter et al., 2013). Thus, these studies demonstrated that AgRP-mediated inhibition of CGRP-positive PBN neurons was necessary for feeding to occur.

The laboratory of Scott Sternson used an optogenetic approach to determine the effects of selectively stimulating AgRP neuron axon terminals in downstream brain regions on food intake behavior. Stimulating AgRP projections in the PVN, BNST, lateral hypothalamus, and paraventricular thalamic nucleus (PVT) resulted in a statistically significant increase in food intake, whereas stimulating projections in the PBN, CeA, and PAG had no significant effect (Atasoy et al., 2012; Betley et al., 2013). Interestingly, using cell-type specific viral tracing tools, Betley et al. (2013) found that individual AgRP neurons tend to preferentially project to individual downstream regions (as opposed to projecting to two or more areas), demonstrating that AgRP neurons form multiple, parallel circuits to regulate feeding behavior.

Obviously, the findings indicating the functional relevance of AgRP neural projections to the PBN from the Palmiter group and the findings indicating importance of AgRP neural projections to the PVN, BNST, lateral hypothalamus (LH), and PVT, as well as previous AgRP neuropeptide injection studies that demonstrate functional importance in the CeA and PVN, need to be reconciled. It is important to note the differences in methodology between studies: while the Palmiter group demonstrated the *necessity* of inhibiting CGRP PBN neurons for feeding, the Sternson group demonstrated the *sufficiency* of other downstream brain regions in causing food intake. AgRP neurons likely orchestrate food intake through several mechanisms, stimulating regions that promote the hedonic, emotional, and craving aspects of hunger, while simultaneously inhibiting mechanisms of satiety and appetite suppression. Thus, stimulating AgRP projections to the PBN may not directly stimulate food intake, but instead may reduce appetite suppression during conditions of relatively low food intake. This hypothesis is supported by previous studies showing that central injection of AgRP overcomes appetite suppression in rodent models of cancer and conditioned taste aversion (Wirth et al., 2002; Joppa et al., 2007). Future experiments should stimulate AgRP neuron projections to the PBN during conditions of visceral malaise to determine if they are sufficient to overcome PBN-induced appetite suppression.

### AgRP Neuron Projections that Mediate Non-Feeding Behaviors

**Figure 2:**
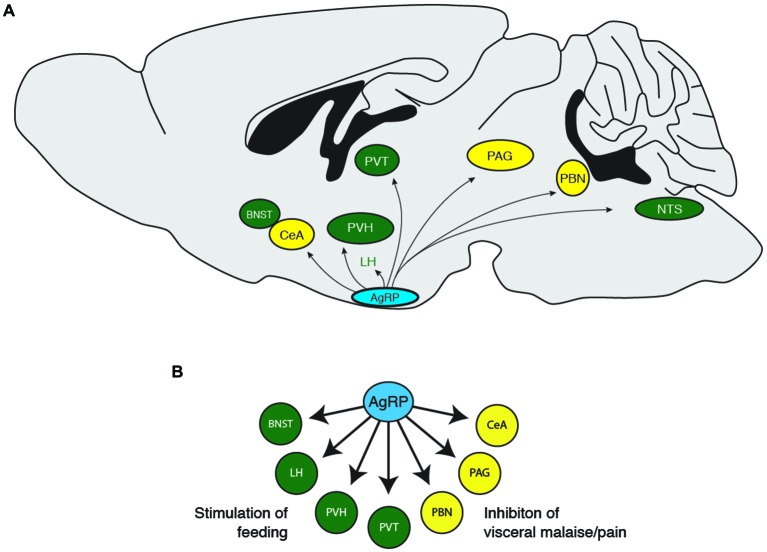
**(A)** Downstream projections from AgRP neurons and **(B)** their potential functional roles in food-seeking behavior. BNST, bed nucleus of the stria terminalis; CeA, central nucleus of the amygdala; LH, lateral hypothalamus; PAG, periaqueductal gray; PBN, parabrachial nucleus; PVH, paraventricular hypothalamus; PVT, paraventricular thalamic nucleus.

During a hungry behavioral state, animals tend to shut down other physiological processes such as growth, reproduction, and nociception, while increasing food exploratory activity and the HPA stress response. Consistently, previous studies demonstrate that central injection of AgRP neuropeptides inhibit puberty and sex hormones (Vulliémoz et al., 2005; Sheffer-Babila et al., 2013), reduce nociception (Bellasio et al., 2003; Bertorelli et al., 2005), increase locomotion (Dietrich et al., 2012), and increase plasma stress hormone concentrations (Dhillo et al., 2002; Xiao et al., 2003). These studies indicate that hunger is about much more than simply increasing food intake, but also mediating other behavioral states to ensure that an organism prioritizes feeding over behaviors that are not immediately essential for survival (Figure 2). Future studies investigating the functional roles of AgRP neuron downstream projections should incorporate behavioral assays in addition to food consumption. Such studies will add to the valuable contributions of the Palmiter, Sternson, and other groups, to show how the AgRP neuron population orchestrates the physiological and behavioral response to hunger.

## The OVLT Orchestrates Water-Seeking Behavior

### OVLT Neurons and Thirst

Like brain arousal systems and food-intake systems, there is probably no single population of neurons that regulates drinking behavior. However, much evidence has focused on the hypothalamic OVLT as a central population of neurons that orchestrates thirst and the physiological response to dehydration (Bourque et al., 1994; McKinley et al., 2006; Bourque, 2008). These neurons are located at the anterior wall of the third ventricle, a circumventricular organ with a relative relaxation of the blood brain barrier (McKinley et al., 2003). Thus, these neurons are in a prime location to sense plasma osmolality, transduce information about fluid hypertonicity into neural activity, and orchestrate the behavioral state of thirst.

Multiple studies demonstrate an increase in OVLT neural activity in response to thirst. OVLT neurons increase expression of Fos, an indirect marker of neural activity, in response to dehydration (McKinley et al., 1994; Ji et al., 2005; Gottlieb et al., 2006; Yoshimura et al., 2013), hypertonic saline (Oldfield et al., 1991; Kovács and Sawchenko, 1993; Bisley et al., 1996; Somponpun et al., 2004; Ho et al., 2007; Miller et al., 2013), and administration of the thirst-generating hormone angiotensin II (McKinley et al., 1992; Oldfield et al., 1994). Electrophysiological recordings *in vivo* and *in vitro* confirm that the rate of OVLT firing increases with the concentration of extracellular fluid osmolality (Sayer et al., 1984; Vivas et al., 1990; Nissen et al., 1993; Ciura and Bourque, 2006), demonstrating that these neurons can directly translate hypertonicity into an increase in neural activity. Complementary studies in humans using functional magnetic resonance imaging demonstrate an increase in OVLT activity in response to hypertonic stimuli (Egan et al., 2003; Morita et al., 2004; Farrell et al., 2011).

Multiple studies also suggest that the OVLT is necessary and sufficient for water-seeking behavior. Loss-of-function studies demonstrate that lesions of the OVLT result in significant deficits in drinking and water-seeking behavior (Buggy and Jonhson, 1977; McKinley et al., 1982, 1999; Thrasher et al., 1982; Thrasher and Keil, 1987; Hochstenbach and Ciriello, 1996). Likewise, gain-of-function studies show that injection of hypertonic saline directly into the OVLT region causes immediate and reversible water-seeking behavior (Andersson, 1971; Buggy et al., 1979).

### OVLT Projections that Mediate Thirst

**Figure 3:**
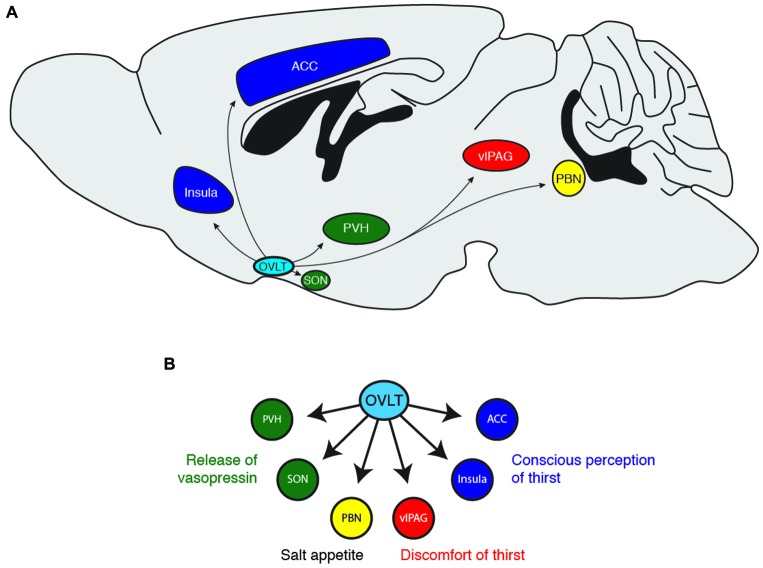
**(A)** Downstream projections from OVLT neurons and **(B)** their potential functional roles in water-seeking behavior. ACC, anterior cingulate cortex; PBN, parabrachial nucleus; PVH, paraventricular hypothalamus; SON, supraoptic nucleus; vlPAG, ventrolateral periaqueductal gray.

How does the OVLT orchestrate the response to dehydration and extracellular hypertonicity? OVLT neurons are known to project to several brain regions (Camacho and Phillips, 1981; Farrell et al., 2011), notably the PVN (Larsen and Mikkelsen, 1995; Morita et al., 2004; Shi et al., 2008), the supraoptic nucleus (SON; Honda et al., 1990; Richard and Bourque, 1992, 1995; Yang et al., 1994; Armstrong et al., 1996; Bourque and Richard, 2001; Morita et al., 2004; Trudel and Bourque, 2010), the insular and anterior cingulate cortex (Egan et al., 2003; Hollis et al., 2008; Farrell et al., 2011), the vlPAG (Uschakov et al., 2009), the PBN (Moga et al., 1990), and the MnPO (Camacho and Phillips, 1981). Each of these regions probably coordinates a distinct organismal response to conserve and seek water (Figure 3). For example, magnocellular neurons in the PVN and SON produce the hormone vasopressin to enhance water reabsorption in the kidneys. Thus, it is likely that OVLT-to-PVN and OVLT-to-SON projections mediate the body’s physiological response to water deficits (Richard and Bourque, 1995; Bourque and Richard, 2001; Shi et al., 2008; Trudel and Bourque, 2010). Projections to the insular and anterior cingulate cortices have been suggested to mediate the conscious perception of thirst (Egan et al., 2003; Hollis et al., 2008), while the vlPAG may mediate the discomfort and “pain” resulting from lack of water (Uschakov et al., 2009). The PBN contains neurons that regulate salt appetite (Geerling and Loewy, 2007; Geerling et al., 2011), and therefore may integrate homeostatic information about osmotic balance to regulate behavioral control of plasma sodium concentration. Interestingly, the MnPO projects to central regions involved in thermoregulation (Uschakov et al., 2007; Nakamura and Morrison, 2008) and arousal (Uschakov et al., 2007), perhaps indicating how information about osmotic balance may be integrated into other animal behaviors. Clearly, more work is necessary to clarify the functional role of these projections and determine how OVLT neurons orchestrate the behavioral state of thirst.

Unlike Hcrt and AgRP neurons, the OVLT does not seem to express a unique genetic marker, hence its name reflects an anatomical location and not expression of a neuropeptide. Unfortunately for neuroscientists, this lack of a genetic marker also presents challenges in the application of spatially precise experimental tools such as optogenetics, pharmacogenetics, or even mouse knockout studies. Future studies should attempt to identify a unique genetic marker for these neurons, as has been accomplished for specific nuclei in other systems (Sanz et al., 2009; Knight et al., 2012; Ekstrand et al., 2014). In the absence of such a marker, perhaps it will be possible to apply genetically encoded neuromodulation tools using Fos promoter-based temporal expression vectors (Reijmers et al., 2007) or projection-based anatomical tools that deliver genes to neurons based on their axonal projection patterns (Gradinaru et al., 2010). Such tools would allow for a thorough characterization of OVLT neurons in osmoregulation and the contribution of each of its efferent targets.

## Conclusion

Clearly, much impressive work over the past two decades has elucidated the role of Hcrt neurons in wakefulness, AgRP neurons in food-seeking behavior, and OVLT neurons in water-seeking behavior. The recent development of genetically encoded neuromodulation tools has greatly advanced our understanding of these systems. Because it is currently unknown if OVLT neurons express a distinct genetic marker, it has been relatively difficult to target these neurons compared with the Hcrt and AgRP systems. Nevertheless, the identification of these systems as neural populations capable of detecting homeostatic signals from the body and orchestrating behavioral states of wakefulness, hunger, and thirst, are well established.

So far, most studies applying optogenetic or DREADD stimulation/inhibition to these neurons have generally employed behavioral assays that examine their global behavioral states. For example, most studies of Hcrt neurons have tested the effects of stimulation/inhibition on EEG/EMG-based parameters of wakefulness, while most studies of AgRP neurons have tested effects on total food intake. Given that these neural populations project diffusely to many brain regions, these cutting-edge methods can now be used to examine other aspects of wakefulness and ingestive behaviors, such as stress, motivation, and organismal physiology.

Recent studies about the role of individual downstream projections from hypothalamic nuclei that “orchestrate” behavior seem to suggest two lessons for future studies: (1) If stimulation of a neural projection (i.e., axon terminals in a downstream region) does not reproduce the effects of stimulation at the cell soma, such a result does not indicate that the neural projection doesn’t play a role in the overall behavior. For example, if stimulating the projections of AgRP neurons to the PAG or PBN does not produce feeding behavior (Betley et al., 2013), these projections likely have other vital functions, such as inhibiting visceral signals of fullness or satiety. Additionally, (2) the projection patterns of these neural populations inform our definitions of what it means to be tired/awake, hungry/full, and thirsty/quenched. For example, we are conditioned to think of hunger as a state of “emptiness”, analogous to an empty signal on a gas tank. However, a hungry behavioral state must engage pathways that increase arousal, motivation, and anxiety while simultaneously suppressing unnecessary behaviors, such as growth and reproduction. Current and future studies of Hcrt, AgRP, and OVLT neurons are exciting because they reveal the complex nature of the behavioral states of wakefulness, hunger, and thirst, and prove that these states are truly more than the sum of their parts.

## Conflict of Interest Statement

The authors declare that the research was conducted in the absence of any commercial or financial relationships that could be construed as a potential conflict of interest.
